# Risk for intracranial hemorrhage in individuals after mild traumatic brain injury who are taking serotonergic antidepressants

**DOI:** 10.3389/fneur.2022.952188

**Published:** 2022-12-07

**Authors:** Harri Isokuortti, Grant L. Iverson, Jussi P. Posti, Ksenia Berghem, Anna-Kerttu Kotilainen, Teemu M. Luoto

**Affiliations:** ^1^Department of Neurosurgery, Helsinki University Hospital and University of Helsinki, Helsinki, Finland; ^2^Department of Physical Medicine and Rehabilitation, Harvard Medical School, Boston, MA, United States; ^3^Department of Physical Medicine and Rehabilitation, Spaulding Rehabilitation Hospital and the Schoen Adams Research Institute at Spaulding Rehabilitation, Charlestown, MA, United States; ^4^Neurocenter, Department of Neurosurgery, and Turku Brain Injury Center, Turku University Hospital and University of Turku, Turku, Finland; ^5^Medical Imaging Centre, Department of Radiology, Tampere University Hospital, Tampere, Finland; ^6^Department of Surgery, Tampere University Hospital and Tampere University, Tampere, Finland; ^7^Department of Neurosurgery, Tampere University Hospital and Tampere University, Tampere, Finland

**Keywords:** antithrombotic medication, anticoagulants, antidepressant agents, intracranial hemorrhages, brain injuries

## Abstract

**Background:**

Serotonergic antidepressants may predispose to bleeding, but little is known of the risk for traumatic intracranial bleeding.

**Methods:**

This was a prospective case-control study of 218 patients with mild traumatic brain injuries (TBI) who were treated at a Finnish tertiary trauma hospital. Injury-related information and clinical findings were prospectively collected in the emergency department. Detailed pre-injury health history was collected from electronic medical records. Information on the use of serotonergic antidepressants was attained from the Finnish national prescription registry. All head CT scans were reviewed by a neuroradiologist based on the Common Data Elements. Cases were patients with traumatic intracranial hemorrhage on head CT. Controls were patients from the same cohort, but without traumatic intracranial lesions on CT. The proportion with traumatic intracranial bleeding for patients on serotonergic antidepressant medication was compared to the proportion for patients not on serotonergic medication.

**Results:**

The study cohort consisted of 24 cases with traumatic intracranial bleeding and 194 injured controls. The median age of the sample was 70 years (interquartile range = 50–83). One fifth (21.6%) of all the patients were taking a serotonergic antidepressant. Of the patients on an antidepressant, 10.6% (5/47) had an acute hemorrhagic lesion compared to 11.1% (19/171) of those who were not on an antidepressant (*p* = 0.927). In the regression analysis, traumatic intracranial hemorrhage was not associated with antidepressant use.

**Conclusion:**

Serotonergic antidepressant use was not associated with an increased risk of traumatic intracranial hemorrhage after a mild TBI. The patients in this relatively small cohort were mostly middle-aged and older adults. These factors limit the generalizability of the results in younger patients with mild TBI.

## Introduction

Head trauma can cause life-threatening traumatic intracranial hemorrhage requiring neurosurgical interventions and specialized critical care. Multiple factors increase the risk for traumatic intracranial bleeding, such as age, high-energy trauma mechanism, coagulopathy, and use of anticoagulants (1–5).

Antidepressants, especially selective serotonin reuptake inhibitors (SSRIs), are frequently used medications. The estimated prevalence of antidepressant use is between 2.7 and 15.7% in the adult population in the European Union and United States (6, 7). Observational studies have found an association between SSRIs and abnormal bleeding (8, 9). This increase in bleeding risk is thought to be due to the effects of SSRIs in the initiation of hemostasis—SSRIs inhibit the role of serotonin in platelet aggregation and directly decrease the platelet adhesion to both collagen and fibrinogen (10–13).

Several studies have also suggested increased risk of intracerebral hemorrhage (ICH) in patients treated with SSRIs (14–17), but this remains unconfirmed (18, 19). The most recent work on pre-ICH SSRI use and ICH risk (20) controlled for multiple covariates, focused on primary ICH, and specifically analyzed SSRI use. In this study, no significant association between pre-ICH SSRI use and ICH risk was found.

Theoretically, serotonergic antidepressants could increase the risk of traumatic intracranial bleeding given the effect of SSRIs on hemostasis. Knowledge of the possible bleeding risks of serotonergic antidepressants in the setting of traumatic brain injury (TBI) is limited. The work by Gaist et al. (21) reported that the use of antidepressants was associated with a higher risk of subdural hemorrhage (both traumatic and non-traumatic), compared to no antidepressant use. The absolute risk was low and appeared to be mainly limited to the first year of treatment. A recently published retrospective study of a cohort of patients seen in the emergency department (ED) following head trauma found a comparable proportion of intracranial bleeding in patients on serotonergic antidepressants and in patients not taking serotonergic antidepressants (22).

The purpose of this study is to investigate the risk for intracranial hemorrhage in patients on serotonergic antidepressant therapy in a prospective sample. We hypothesized that the use of serotonergic antidepressants would not be associated with an increased risk for intracranial hemorrhages following traumatic brain injury.

## Materials and methods

### Study setting and ethics

The patients were originally recruited for a validation study (23) to investigate whether serum S100B could be used to reduce CT scans after head trauma as part of the newer Scandinavian guideline for Initial Management of Minimal, Mild, and Moderate Head Injuries in Adults (24). The guidelines are designed for patients with acute (within 24 h from injury) TBI and for detecting important intracranial injuries. Using the guideline, mild TBIs are categorized in low, medium, and high-risk groups depending on certain risk factors (including antiplatelet or anticoagulation medication).

The Tampere University Hospital is the main trauma center and only neurosurgical referral hospital in the hospital district. The ED provides health services for a joint municipal authority of 22 municipalities (both urban and rural) with a total of ~470,000 residents. In this study, the minimum criteria for TBI were defined as follows: either blunt injury to the head or acceleration/deceleration type injury resulting in an initial Glasgow Coma Scale (GCS) score of < 15, witnessed loss of consciousness, disorientation, or amnesia (24).

This study was approved by the Ethics Committee of Pirkanmaa Hospital District, Tampere, Finland (ethical code: R15045). Institutional ethics and research board approval was obtained. All the patients included provided written informed consent according to the Declaration of Helsinki.

### Study sample

The participants were enrolled during a 1-year period between November 2015 and November 2016. A cohort of 3,067 adult (≥18 years) patients with head injuries were treated in the ED during that time. Written consent was obtained from 325 patients, 218 of these had a mild TBI (GCS of 14–15 on admission) and a head CT-scan and included in the present study. Head CT was performed according to the most recent Scandinavian head injury guidelines (24). Injury-related information and clinical findings were collected in the ED. Detailed pre-injury health history was collected from the electronic medical records.

### Cases and controls

The event was defined as a traumatic hemorrhagic lesion in the head CT scan performed in the ED. Patients selected from the same cohort but without intracranial bleeding served as controls. The exposure variable was the use of serotonergic antidepressants. Head CT lesions that were considered traumatic were epidural hematoma, acute subdural hematoma, contusion, traumatic subarachnoid hemorrhage, traumatic intracerebral hemorrhage, and traumatic intraventricular hemorrhage. There was no attempt to match cases and controls on any variables.

### Medication data

The information on the current use of antidepressants and antithrombotic medication was attained from the national drug prescription registry maintained by the Social Insurance Institution of Finland, Helsinki, Finland. The dose and duration of use were not available. Antidepressants that were considered serotonergic and are in use in Finland were: (i) citalopram, (ii) escitalopram, (iii) sertraline, (iv) fluoxetine, (v) paroxetine, (vi) venlafaxine, (vii) duloxetine, (vii) vortioxetine, (viii) amitriptyline, (ix) nortriptyline, (x) doxepin, and (xi) trimipramine. Drugs were further grouped into SSRIs, serotonin-norepinephrine reuptake inhibitors (SNRIs), and tricyclic antidepressants (TCAs) based on their structure and pharmacodynamic properties. Vortioxetine was placed to the SNRI group based on its pharmacological features.

Antithrombotic medication use was also recorded. Antiplatelet medications included acetylsalicylic acid (ASA), ASA-dipyridamole, dipyridamole, ticlodipine, clopidogrel, prasugrel, and ticagrelor. Anticoagulant medication included warfarin, apixaban, dabigatran, edoxaban, and rivaroxaban, and low-molecular weight heparins (LMWH). The antithrombotic medication history was obtained by interviewing the patient at enrollment.

### Imaging

All of the head CT scans were interpreted by a board certified neuroradiologist and systematically coded using the National Institutes of Health/National Institute of Neurological Disorders and Stroke (NIH-NINDS) Traumatic Brain Injury (TBI) Imaging Common Data Elements (25). The median time from the injury to the CT scan was 5.1 h (interquartile range 2.8–10.2 h). To assess the progression in the hemorrhagic lesions, we obtained the data from the follow-up CT scans within the next seven days from the index injury.

### Statistical analyses

Dichotomous variables were compared using the chi-square test for proportions and continuous variables using the Mann–Whitney *U*-test. Logistic regression modeling was performed to estimate the odds ratios (ORs) and 95% confidence intervals (CI) of acute intracranial hemorrhage in patients on serotonergic antidepressants while adjusting for GCS and the use of any antithrombotic medication (antiplatelet and/or anticoagulant medication, dichotomized yes/no). In the model, the GCS was dichotomized to 15 and 14. The regression model covariates were selected based on their clinical relevance in relation to the intracranial hemorrhage risk. The level of statistical significance was set at 5%. SPSS Statistics, versions 24–27 (IBM Corp., Armonk, NY, USA) were used for data analyses. STROBE guidelines were followed (26).

## Results

The characteristics of the study sample are presented in Table 1, stratified by the exposure to the serotonergic antidepressants. The same characteristics stratified by the case-control status (i.e., presence of an acute hemorrhagic lesion or not) is presented in the Table 2. The median age of the patients was 70 years. The most common injury mechanism was a ground-level fall. Antithrombotic medication was used by 47.2%. The most common anticoagulant was warfarin, and the most used antiplatelet medication was ASA.

**Table 1:** Characteristics of the study patients.

**Variable**	**All patients** ***n*** = **218**		**On serotonergic antidepressants** ***n*** = **47**		**Not on serotonergic antidepressants** ***n*** = **171**		*p*-Value^*^
Age (years)	Md = 70	IQR = 50–83	Md = 63	IQR = 43–83	Md = 71	IQR = 51–83	0.478
	*n*	%	*n*	%	*n*	%	
Men	101	47	31	66.0	85	49.7	0.048
Women	116	53	16	34.0	86	50.3	
Diseases of the circulatory system (I00-99)	148	67.9	33	70.2	115	67.3	0.700
Mental and behavioral disorders (F00-99)	90	41.3	25	53.2	65	38.0	0.159
Diseases of the nervous system (G00-99)	82	37.6	23	48.9	59	34.5	0.070
**Injury mechanism**							
Unknown	2	0.9	0	0.0	2	1.2	
Car accident	3	1.4	0	0.0	3	1.8	
Ground-level fall	158	72.5	35	74.5	123	71.9	0.730
Motorcycle accident	1	0.5	0	0.0	1	0.6	
Bicycle accident	5	2.3	0	0.0	5	2.9	
Fall from a height	26	11.9	7	14.9	19	11.1	0.479
Sports	8	3.7	1	2.1	7	4.1	
Violence	8	3.7	1	2.1	7	4.1	
Other, not specified	7	3.2	3	6.3	4	2.3	
**Anticoagulant medication use**	64	29.4	12	25.5	52	30.4	0.515
Warfarin	59	27.1	10	21.3	49	28.7	
Apixaban	1	0.5	1	2.1	0	0.0	
Dabigatran	3	1.4	1	2.1	2	1.1	
Rivaroxaban	1	0.5	0	0.0	1	0.6	
**Antiplatelet medication use**	44	20.2	10	21.3	34	19.9	0.833
Acetylsalicylic acid	35	16.1	6	12.8	29	17.0	
Acetylsalicylic acid-dipyridamole	4	1.8	2	4.3	2	1.1	
Clopidogrel	5	2.3	2	4.3	3	1.8	
Ticagrelor	1	0.5	0	0.0	1	0.6	
Acetylsalicylic acid and clopidogrel	1	0.5	0	0.0	1	0.6	
**Glasgow Coma Scale score**							
15	208	95.4	165	96.5	43	91.5	0.147
14	10	4.6	6	3.5	4	8.5	
Loss of consciousness, witnessed	37	17.0	8	17.0	29	17.0	0.992
Post-traumatic amnesia	92	42.2	20	42.6	72	42.1	0.956
Focal neurological deficit	24	11.0	6	12.8	18	10.5	0.664
Hemorrhagic lesion on head CT	24	11.0	5	10.6	19	11.1	0.927

^*^Dichotomous variables chi square test, continuous variables Mann–Whitney U-test.

CT, computed tomography; Md, median; IQR, interquartile range.

**Table 2:** Characteristics of the study patients stratified by the head CT findings.

**Variable**	**No hemorrhagic lesions (*n* = 194)**	**Hemorrhagic lesion (*n* = 24)**
Age (years)	Md = 69, IQR = 48–82	Md = 78, IQR = 68–85
Women	103 (53.1%)	13 (54.2%)
**SSRI**	28 (14.4%)	3 (12.5%)
Fluoxetine	1 (0.5%)	0 (0.0%)
Citalopram	8 (4.1%)	1 (4.2%)
Escitalopram	16 (8.2%)	1 (4.2%)
Sertraline	3 (1.5%)	1 (4.2%)
Paroxetine	2 (1.0%)	0 (0.0%)
**SNRI**	9 (4.6%)	0 (0.0%)
Duloxetine	5 (2.6%)	0 (0.0%)
Venlafaxine	5 (2.6%)	0 (0.0%)
**TCA**	11 (5.7%)	2 (8.3%)
Amitriptyline	9 (4.6%)	1 (4.2%)
Doxepin	0 (0.0%)	0 (0.0%)
Nortriptyline	3 (1.5%)	0 (0.0%)
Trimipramine	0 (0.0%)	1 (4.2%)
**Other**		
Vortioxetine	1 (0.5%)	0 (0.0%)
Any serotonergic medication	42 (21.6%)	5 (20.8%)
**Antiplatelet use**	36 (18.6%)	8 (33.3%)
Aspirin	29 (14.9%)	6 (25.0%)
Aspirin-dipyridamole	3 (1.5%)	1 (4.2%)
Tigagrelor	0 (0.0%)	1 (4.2%)
Clopidogrel	4 (2.1%)	1 (4.2%)
**Anticoagulant use**	54 (27.8%)	10 (41.7%)
Apixaban	1 (0.5%)	0 (0.0%)
Warfarin	50 (25.8%)	9 (37.5%)
Dabigatran	2 (1.0%)	1 (4.2%)
Rivaroxaban	1 (0.5%)	0 (0.0%)
**Severity indicators**		
Posttraumatic amnesia	78 (40.2%)	14 (58.3%)
Loss of consciousness	30 (15.5%)	7 (29.2%)
Focal neurological deficits	21 (10.8%)	3 (12.5%)
**Glasgow Coma Scale**		
15	188 (96.9%)	20 (83.3%)
14	6 (3.1%)	4 (16.7%)
**Mechanism of injury**		
Bicycle accident	5 (2.6%)	0 (0.0%)
Car accident	3 (1.5%)	0 (0.0%)
Fall from a height	21 (10.8%)	5 (20.8%)
Ground level fall	139 (71.6%)	19 (79.2%)
Motorcycle accident	1 (0.5%)	0 (0.0%)
Sport injury	8 (4.1%)	0 (0.0%)
Violence	8 (4.1%)	0 (0.0%)
Unknown	2 (1.0%)	0 (0.0%)
Other	7 (3.6%)	0 (0.0%)
**Health problems**		
Diseases of the nervous system (G00-G99)	77 (39.7%)	5 (20.8%)
Mental and behavioral disorders (F00-F99)	82 (42.3%)	8 (33.3%)
Diseases of the circulatory system (I00-I99)	128 (66.0%)	20 (83.3%)

Md, median; IQR, inter-quartile range; SSRI, selective serotonin reuptake inhibitor; SNRI, serotonin and norepinephrine reuptake inhibitor; TCA, tricyclic antidepressant.

The use of serotonergic antidepressants is presented in Table 3. One fifth (21.6%) of the patients were taking a serotonergic antidepressant. SSRIs were the most commonly used antidepressants (14.2%), and the most common agent was escitalopram (7.8%).

**Table 3:** Use of serotonergic antidepressants stratified by the head CT finding and antithrombotic medication use.

	**Whole sample** ***n*** = **218**		**On antithrombotic medication** ***n*** = **103**		**Hemorrhagic lesion visible on head** **CT** ***n*** = **24**		**Head CT-negative** ***n*** = **194**		**Head CT-positive and** **on antithrombotic** **medication** ***n*** = **16**		**Head CT-negative and** **on antithrombotic** **medication** ***n*** = **87**	
	***n***	**%**	***n***	**%**	***n***	**%**	***n***	**%**	***n***	**%**	***n***	**%**
**SSRI**	31	14.2	16	15.5	3	12.5	28	14.4	3	18.8	13	14.9
Fluoxetine	1	0.5	0	0.0	0	0.0	1	0.5	0	0.0	0	0.0
Paroxetine	2	0.9	0	0.0	0	0.0	2	1.0	0	0.0	0	0.0
Sertraline	4	1.8	1	6.3	1	4.2	3	1.5	1	6.3	1	1.1
Citalopram	9	4.1	6	5.8	1	4.2	8	4.1	1	6.3	5	5.7
Escitalopram	17	7.8	8	7.8	1	4.2	16	8.2	1	6.3	7	8.0
**SNRI**	9	4.1	3	2.9	0	0.0	9	4.6	0	0.0	3	3.4
Duloxetine	5	2.3	3	2.9	0	0.0	5	2.6	0	0.0	3	3.4
Venlafaxine	5	2.3	0	0.0	0	0.0	5	2.6	0	0.0	0	0.0
**TCA**	13	6.0	4	3.9	2	8.3	11	5.7	1	6.3	3	3.4
Amitriptyline	10	4.6	3	2.9	1	4.2	9	4.6	0	0.0	3	3.4
Doxepine	0	0.0	0	0.0	0	0.0	0	0.0	0	0.0	0	0.0
Nortriptyline	3	1.4	1	1.0	0	0.0	3	1.5	0	0.0	1	1.1
Trimipramine	1	0.5	1	1.0	1	4.2	0	0.0	1	6.3	0	0.0
**Other**
Vortioxetine	1	0.5	0	0.0	0	0.0	1	0.5	0	0.0	0	0.0
Any serotonergic medication^*^	47	21.6	22	21.4	5	20.8	42	21.6	4	25.0	18	20.7

* Some patients were taking two or more antidepressant medication simultaneously.

CT, computed tomography; SSRI, selective serotonin reuptake inhibitor; SNRI, serotonin-norepinephrine reuptake inhibitor; TCA, tricyclic antidepressant.

**Figure 1:**
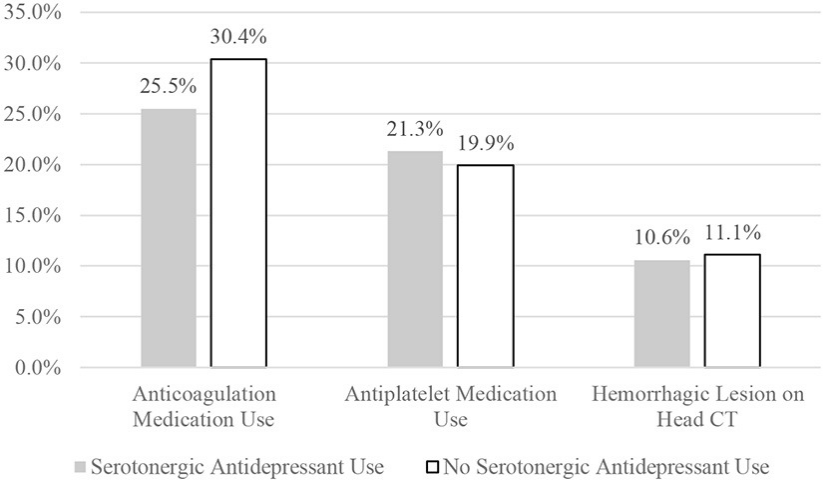
Comparing the two groups on medication use and hemorrhagic lesions.

Of the patients on an antidepressant, 10.6% (5/47) had an acute hemorrhagic lesion compared to 11.1% (19/171) of those who were not on an antidepressant (χ^2^ (1) = 0.008, *p* = 0.927, OR = 1.0, 95% CI 0.3–2.7; see Figure 1). Hemorrhagic CT lesions stratified by medication use are presented in detail in Table 4. Cross-tabulations of individual serotonergic antidepressants and hemorrhagic CT lesions are presented in Table 5.

**Table 4:** Hemorrhagic CT lesions stratified by groups.

	**Sample size**	**Hemorrhagic lesion on** **head CT**		*p*-Value^*^
		***f***	**%**	
Total sample	218	24	11.0	
Serotonergic antidepressant	47	5	10.6	0.927
No serotonergic antidepressant	171	19	11.1	
Antithrombotics	103	16	15.5	0.043
No antithrombotics	115	8	7.0	
Serotonergic antidepressant and antithrombotic	22	4	18.2	0.142
No serotonergic antidepressant and no antithrombotic	90	7	7.8	

f, frequency; %, percentage; CT, computed tomography.

* Chi square test.

**Table 5:** Cross-tabulation of individual serotonergic antidepressants and hemorrhagic CT lesions.

**Antidepressant (patients on this medication)**	**EDH**	**SDH (acute)**	**SAH**	**Contusion**	**ICH**	**IVH**	**Any hemorrhagic lesion**
**SSRIs (31)**	0	2	2	2	1	0	3
Fluoxetine (1)	0	0	0	0	0	0	0
Paroxetine (2)	0	0	0	0	0	0	0
Sertraline (4)	0	1	1	1	1	0	1
Citalopram (9)	0	0	1	1	0	0	1
Escitalopram (17)	0	1	0	0	0	0	1
**SNRIs (9)**	0	0	0	0	0	0	0
Duloxetine (5)	0	0	0	0	0	0	0
Venlafaxine (5)	0	0	0	0	0	0	0
**Tricyclics and tetracyclics (13)**	0	1	2	0	0	0	2
Amitriptyline (10)	0	0	1	0	0	0	1
Doxepin (0)	0	0	0	0	0	0	0
Nortriptyline (3)	0	0	0	0	0	0	0
Trimipramine (1)	0	1	1	0	0	0	1
**Other antidepressants (1)**	0	0	0	0	0	0	0
Vortioxetine (1)	0	0	0	0	0	0	0
**Total**	0	3	4	2	1	0	5

Each cell represents the number of cases.

EDH, epidural hematoma; SDH, subdural hematoma; SAH, subarachnoid hemorrhage; ICH, intracerebral hemorrhage; IVH, intraventricular hemorrhage; SSRI, selective serotonin reuptake inhibitor; SNRI, serotonin-norepinephrine reuptake inhibitor; TCA, tricyclic antidepressant.

Three patients had multiple traumatic lesions on head CT.

In the adjusted logistic regression model, the use of serotonergic antidepressant was not associated with traumatic intracranial hemorrhages (OR = 0.8, 95% CI = 0.3–2.5, *p* = 0.733). GCS less than 15 was associated with bleeding (OR = 7.6, 95% CI = 1.8–31.3, *p* = 0.005). The use of antithrombotic medication was also associated with intracranial hemorrhages (OR = 2.7, 95% CI = 1.1–6.8, *p* = 0.036).

Of the 24 patients with a hemorrhagic lesion on CT, eight patients were reimaged within a week after the initial CT scan. There were five patients whose follow-up CT showed progression in the intracranial bleeding. Only one of these patients was taking a serotonergic antidepressant. This patient was on sertraline and warfarin. He fell and suffered an acute subdural hematoma, traumatic subarachnoid bleeding, brain contusion, and a skull fracture. The effects of warfarin were reversed, and he did not need any neurosurgical procedures.

## Discussion

The purpose of this study is to investigate the risk for intracranial hemorrhage in patients on serotonergic antidepressant therapy in a prospective sample. We hypothesized that the use of serotonergic antidepressants would not be associated with an increased risk for intracranial hemorrhages following head injury. In this study, there was not an association between traumatic intracranial bleeds and use of serotonergic antidepressants. Specifically, the percentages with traumatic hemorrhagic lesions were similar in patients taking antidepressants compared to patients not taking antidepressants. These results are important given the high incidence of head injuries, the widespread use of serotonergic antidepressants, and the prior reports of serotonergic antidepressant related systemic bleeding complications.

Prior literature on the effects of antidepressant and traumatic intracranial bleeding is scarce. Ibañez Pérez De La Blanca and colleagues concluded that SSRIs and/or benzodiazepines were protective against CT-positive intracranial lesions (27). In a Danish registry study, the use of SSRI and non-SSRI antidepressants was associated with a higher risk of acute and chronic subdural hematomas, compared to no antidepressant use (21). The absolute risk was low, and it appeared to be mainly limited to the first year of treatment.

The results of the present study are in line with a larger retrospective study that found no association between traumatic intracranial bleeds and use of serotonergic antidepressants (22). In line with the literature, antithrombotic agents were associated with an increased risk of CT-positive traumatic intracranial hemorrhage. Our current findings have important clinical implications for the acute management of patients with head injuries. In the emergency assessment of these patients, the risk of intracranial hemorrhagic complications is of paramount importance. Age, preexisting diseases, anticoagulation, and injury characteristics influence the risk of hemorrhage as shown in numerous large-scale studies (1, 2, 4, 5). These factors predict only partly the risk for intracranial hemorrhage and the effect of other medications (e.g., serotonergic antidepressants) affecting the hemostasis has not been thoroughly studied. Decision-making on initial emergency head CT scanning, need for in-hospital monitoring, stratification for monitoring strategies, administration of prothrombotic agents to correct coagulation, and the need for repeat head CT imaging is largely based on the presumed overall risk of intracranial hemorrhagic complications.

This study has several strengths. Adult patients from all age groups in varying health conditions were included, although our sample includes a large percentage of older adults. The medication use of each patient was reviewed thoroughly. It is well-known that adherence to long-term medication is often poor, with adherence rates averaging around 50% (28) and Finland is no exception in this regard (29). However, the Finnish National medication register has been shown to be reliable for estimating the amount and duration of medication use as well as secondary medication adherence (30). All CT findings, including hemorrhagic and other traumatic lesions, were systematically coded. All the patients were treated in the same ED and the CT scans were interpreted by a neuroradiologist.

There are also important limitations in this study. The study sample is relatively small and the number of patients taking serotonergic antidepressants was small—thus the study has limited power for detecting small effects. Readers are encouraged to examine the effect sizes and their corresponding confidence intervals. We could not determine how representative our sample was compared to all patients presenting to the ED. The patients in this cohort had predominantly very mild brain injuries and were relatively old. Because the cohort was relatively small, selection bias may have affected the results. These factors limit the generalizability of the results in younger patients with mild traumatic brain injury and all patients with more severe injuries. Actual use of serotonergic antidepressants was not separately confirmed, only the data on prescriptions were used. It is possible that some patients did not take the medication as we assumed. Unfortunately, we were unable to collect data on the exact dosage of the antidepressant medication, or duration of use. As noted above, there is some uncertainty about the use of ASA, because this drug is available as over the counter medication in Finland. The patient enrollment was performed by on-call physicians working in the ED which reflects the quite low enrollment rate. Detailed reasons for patient exclusion/non-enrollment were not collected due to the pragmatic enrollment design. There were few users of direct oral anticoagulants because their use was uncommon during the period when most data were collected. Matching of the subjects was not possible because of the small study sample and there was no external control group available.

Future high-level observational studies with adequate sample sizes might allow better estimation of the potential risk of SSRI use and traumatic intracranial hemorrhage. The results of this study suggest that the use of serotonergic antidepressants in patients with mild TBIs does not warrant special precautions.

## Data availability statement

The statistical analyses and underlying data supporting the conclusions of this article will be made available by the authors to qualified researchers for research purposes, without undue reservation.

## Ethics statement

The studies involving human participants were reviewed and approved by Ethics Committee of Pirkanmaa Hospital District, Tampere, Finland (ethical code: R15045). The patients/participants provided their written informed consent to participate in this study.

## Author contributions

HI wrote the statistical analysis plan, cleaned and analyzed the data, and drafted and revised the paper. GI and JP contributed to drafting and revising the paper. KB collected the imaging data and contributed to drafting and revising the paper. A-KK collected the medication data and contributed to drafting and revising the paper. TL wrote the statistical analysis plan, monitored data collection for the whole trial, enrolled patients to the study, and contributed to drafting and revising the paper. All of the authors have approved the final version.
